# Review of the Usefulness of Various Rotational Seismometers with Laboratory Results of Fibre-Optic Ones Tested for Engineering Applications

**DOI:** 10.3390/s16122161

**Published:** 2016-12-16

**Authors:** Leszek R. Jaroszewicz, Anna Kurzych, Zbigniew Krajewski, Paweł Marć, Jerzy K. Kowalski, Piotr Bobra, Zbigniew Zembaty, Bartosz Sakowicz, Robert Jankowski

**Affiliations:** 1 Faculty of Advanced Technologies and Chemistry, Military University of Technology, 2 gen. Sylwestra Kaliskiego St., Warsaw 00-908, Poland; jarosz@wat.edu.pl (L.R.J.); zbigniew.krajewski@wat.edu.pl (Z.K.); pawel.marc@wat.edu.pl (P.M.); 2 m-Soft Ltd., 9-4 Sotta Sokoła St., Warsaw 02-790, Poland; jkk@m-soft.pl; 3 Faculty of Civil Engineering and Architecture, Opole University of Technology, 48 Katowicka St., Opole 45-061, Poland; p.bobra@po.opole.pl (P.B.); z.zembaty@po.opole.pl (Z.Z.); 4 Department of Microelectronics and Computer Science, Lodz University of Technology, 221/223 Wólczańska St., Lodz 90-924, Poland; sakowicz@dmcs.pl; 5 Faculty of Civil and Environmental Engineering, Gdansk University of Technology, 11/12 Gabriela Narutowicza St., Gdansk 80-233, Poland; jankowr@pg.gda.pl

**Keywords:** fibre-optic interferometric sensor, rotational seismometer, seismological investigation, strong motion seismology, earthquakes, shaking table

## Abstract

Starting with descriptions of rotational seismology, areas of interest and historical field measurements, the fundamental requirements for rotational seismometers for seismological and engineering application are formulated. On the above basis, a review of all existing rotational seismometers is presented with a description of the principles of their operation as well as possibilities to fulfill formulated requirements. This review includes mechanical, acoustical, electrochemical and optical devices and shows that the last of these types are the most promising. It is shown that optical rotational seismometer based on the ring-laser gyroscope concept is the best for seismological applications, whereas systems based on fiber-optic gyroscopes demonstrate parameters which are also required for engineering applications. Laboratory results of the Fibre-Optic System for Rotational Events & Phenomena Monitoring using a small 1-D shaking table modified to generate rotational excitations are presented. The harmonic and time-history tests demonstrate its usefulness for recording rotational motions with rates up to 0.25 rad/s.

## 1. Introduction

Recently, there has been increasing interest in rotational ground motion measurements. It is believed that rotational signals may contain additional valuable information for studying wave propagation; in addition, rotational ground motion may be important in the excitations of certain engineering structures. According to the introduction to a special issue of the Bulletin of the Seismological Society of America, [1] rotational seismology has become an emerging field for the study of all aspects of rotational ground motion induced by earthquakes, explosions, and ambient vibrations. This domain has attracted the attention of researchers from a wide range of geophysical disciplines, including broadband seismology, strong-motion seismology [2], earthquake engineering including seismic behaviour of irregular and complex civil structures [3,4], earthquake physics [5,6], seismic instrumentation [7], seismic hazards [8], seismotectonics [9], geodesy [10], and from physicists using Earth-based observatories for detecting gravitational waves generated by astronomical sources [11,12].

The likely rotational effects of an earthquake wave, together with the rotations caused by a soil-structure interaction, have been observed for centuries; this is shown in the summary by Kozák [13], in which an image of a rotated obelisk after the 1783 Calabria earthquake is cited as the first illustration of this phenomena. The physical description of earthquake rotational effects is based on two classes of rotational seismic models [14,15]. The first class includes historic models, as defined by Mallet [16] in the mid-nineteenth century, and based on the rotation of bodies with respect to their underlying structures. The second class is derived from recent progress in theoretical studies on micromorphic and asymmetric theories of continuum mechanics as well as in nonlinear physics; an overview of these can be found in monographs by Teisseyre et al. [5,6]. In this field, both theoretical [17,18] and experimental evidence [19] regarding the existence of rotational seismic waves should be considered.

Similarly, recent progress in the structural heath monitoring of civil engineering structures has prompted scientists to investigate the existence of rotations in structural responses to any type of excitations. However, the primary interests of researchers of rotational seismic engineering are associated mainly with formulating additional seismic loads on structures in terms of the rotational seismic excitations. While building responses to translational motion has been thoroughly investigated and implemented into design codes of practice, the study of building response to rotational motions is a relatively new field [20]. This is because the engineering importance of the rotational components of strong seismic ground motion was noted much later than the translational seismic effects [21,22]. From an engineering standpoint, this rotation may be responsible for damage in high-rise buildings [23] and in those structures where the soil-structure interaction effects are expected to be significant [24,25].

Early attempts towards practical studies measuring rotational ground motions can be found in pioneering works from several countries. More than a century ago, Galitzin [26] suggested using two identical pendulums installed on different sides of the same rotational axis for separate measurements of rotational and translational motion. This idea was later used in an instrument designed for the registration of strong ground motion [27] as well as in a system of azimuthal arrays of rotational seismographs for rock bursts in a nearby mine [28]. Another example of an early attempt to measure rotation was the construction of a gyroscopic seismometer which was used to measure a static displacement of <10^−3^ m and a tilt of <0.5 × 10^−6^ rad at La Jolla, California, during the Borrego Mountain earthquake on 9 April 1968 (magnitude 6.5) [29]. Early efforts also included studies of explosions using seismological sensors to directly measure point rotations after nuclear explosions [30], as well as commercial rotational sensors based on microelectro-mechanical systems (MEMS) for identifying significant near-field rotational motions from a one-kiloton explosion [31]. Finally, it should be noted that rotations of and strains in the ground in the responses of structures have been indirectly deduced from accelerometer arrays using methods valid for seismic waves with wavelengths longer than the distances between sensors [32,33,34,35,36,37,38,39].

In Section 2 of this paper, we formulate the fundamental requirements for a rotational seismometer regarding the two main areas of interest described above, i.e., for seismological and engineering applications. The subsequent two sections contain descriptions of all the main types of rotational seismometers with a discussion of their principles of operation and a comparison of their fundamental parameters. These devices can be divided into two groups of sensors: rotational sensors based on classical seismometers which detect rotation in an indirect way, such as mechanical or acoustical types, described in Section 3, and rotational sensors which detect rotation directly, such as the electrochemical, mechanical and optical types (Section 4). These descriptions include a discussion of how far the requirements formulated in Section 2 for seismological and engineering areas of application are fulfilled, and show that the optical type of rotational seismometer, and particularly the type based on a fibre-optic gyroscope (FOG), is the most promising device for future research and application in rotational seismology. On the basis of this conclusion, Section 5 presents the results of a laboratory investigation of the fibre-optic system for rotational events and phenomena monitoring (FOSREM) on a small one-dimensional shaking table modified to generate rotational excitations; these results confirm that FOSREM contains the parameters required for rotational seismology. Finally, Section 6 presents the main conclusions.

## 2. Fundamental Requirements for Rotational Seismometers Depending on Area of Interest

**Figure 1:**
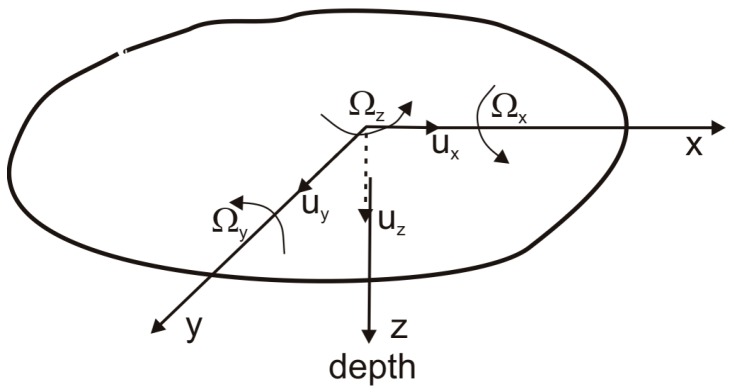
Sketch showing translational and rotational directions on the ground surface.

Traditionally, ground motion measurements in seismology are carried out along one vertical and two perpendicular horizontal axes. However, each point on the ground surface can be associated with six degrees of freedom (Figure 1), that is, the three translations *u*_x_(*t*), *u*_y_(*t*) and *u*_z_(*t*) along the *x*, *y* and *z* axes and three rotations *Ω_x_*, *Ω_y_* and *Ω_z_* about the above axes. Despite the need for experimental evidence described in the previous section, these rotations were not measured for many years, due to a lack of appropriate measurement techniques and the sceptical approach of researchers towards the presence and/or importance of these types of recordings.

Currently, practical studies of rotational events or phenomena are at an early stage, for two reasons. Firstly, the classical seismometer, a device sensitive to linear displacement, velocity or acceleration, cannot detect rotational motions [7], and thus a new class of devices is required. Secondly, the requirements for rotational motion sensors for seismological and engineering applications have not been formulated or agreed upon.

Regarding seismological applications, the following requirements were proposed by Bernauer et al. in 2012 [40]: (1) sensors need to be effectively insensitive to linear motion, or at any time, independent measurement of linear and rotational motions must be possible; (2) for the installation of networks of temporary stations, the instrument needs to be small and stable with respect to ambient conditions, including changes of temperature; (3) similarly, the electrical power supply should be easily managed using batteries, at least in combination with solar panels or fuel cells; (4) a useful instrument for weak motion seismology needs to be able to measure amplitudes on the order of 10^−7^ rad/s at periods of between 10 and 100 s. These four requirements for seismological applications will be referred to as S1 to S4 respectively in the remainder of this paper.

For engineering applications, the situation is more complicated, since the requirements are not specified a priori in the literature. Data on the biggest measured rotation is found to be equal to 3.8 × 10^−3^ rad/s using a high-pass filter 0.2–30 Hz [31]. A vertical-axes peak rotation rate published for a set of data from an earthquake by Takeo [41] was nearly 5 × 10^−3^ rad/s; however, it is likely that expected amplitudes and frequency ranges are much higher, and may fall into the rad/s range [42]. Recent work on collection of data from ground rotations at the surface measuring station located in the mining area of the Ziemowit coal mine in the Upper Silesian Coal Basin in Poland has identified rotation with magnitudes of up to 2.7 and respective rotation rates of up to 0.5 × 10^−3^ rad/s; however, rockbursts with magnitudes exceeding 4 are expected in this area within a few more years [43]. For these reasons, the requirements for rotational seismometer in engineering applications should in the authors’ opinion be as follows: (1–3) the same requirements as for seismological applications (S1–S3); (4) a useful instrument needs to be able to measure amplitudes on the order of a few rad/s at a frequency range of between 10^−2^ and 100 Hz, that is, even higher than that for engineering strong motion seismology [44]. In the remainder of this paper, these four requirements for engineering applications will be referred to as E1 to E4 respectively.

Finally it should be underlined that the rotational sensor used in field applications should be in the form of a seismograph, an instrument that detects and records rotational motions along with timing information. According to the glossary presented by Lee [45], this consists of a rotational seismometer, a precise time source and a recording device; however, in this paper the primary focus will be on the rotational seismometer. A careful analysis of other seismograph components can be found in a book by Havskov and Alguacil [7].

## 3. Rotational Seismometers for Indirect Measurement of Rotation

**Figure 2:**
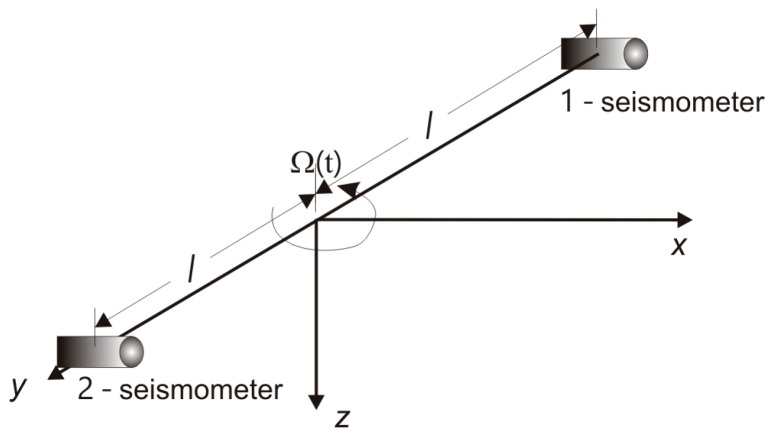
Principle of operation of a rotational seismometer for indirect measurement; sensors 1 and 2 are velocity seismometers.

This type of rotational seismometer uses a pair of standard seismic sensors (pendulums or geophones) oriented parallel to a chosen axis (for instance the *x* axis) and rigidly mounted at a distance *l* along the perpendicular axis, shown as the *y* axis in Figure 2. The rotation rate *Ω_z_* in radians per second around the *z* axis is [7]:

(1) $$\Omega_{z}=\frac{\partial v_{x}}{\partial y}\approx \frac{v_{2}-v_{1}}{l},$$

where *v*_1_ and *v*_2_ are the velocities measured by sensors 1 and 2 along the *x* axis.

### 3.1. Rotational Seismometer Using a Pair of Classical Pendulum Seismometers

**Figure 3:**
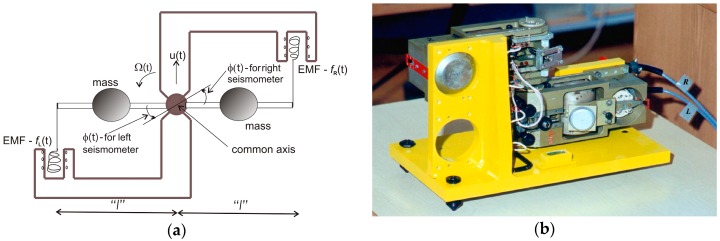
The TAPS rotational seismometer: (**a**) scheme [50]; (**b**) general view. φ(t) is the angle of rotation for a given pendulum.

Teisseyre and Nagahama [46] constructed a practical example of this type of system using two antiparallel pendulum seismometers (SM-3 type, ROTOR International Ltd., Kursk, Russia), known as TAPS; these are positioned on a common axis and connected in parallel, but with opposite orientations, as shown in Figure 3. In the case of ground motion involving a displacement *u*(*t*) and only vertical rotation *Ω_z_* = *Ω*(*t*), the electromotive force (EMF) *f*(*t*) recorded by each SM-3 contains a component of displacement ±*u* and rotational motion *Ω* multiplied by the proper length of the pendulum *l* [46]:

(2) $$f_{L,R}\left(t\right)=\pm u\left(t\right)+l\cdot \Omega \left(t\right),$$

where the signs “+” and “−” represent right (R) and left (L) seismometers respectively. In the case of two identical seismometers, the rotational and translational components can be obtained from the sum and difference of two recorded signals respectively as:

(3) $$\Omega \left(t\right)=\frac{1}{2l}[f_{R}\left(t\right)+f_{L}\left(t\right)]\;\mathrm{and}\;u\left(t\right)=\frac{1}{2l}[f_{R}\left(t\right)-f_{L}\left(t\right)].$$

The fact that the rotational velocity is calculated as the sum of measured signals is typically the reason for difficulties in this measurement. For identical seismometers, this sum is precisely proportional to the rotation velocity. However, in practice the pendulums never are identical, and this implies a difference in the measured signals of an order of magnitude lower than the magnitude of the component signal when the signals are intense; for weak signals this difference may be another order of magnitude lower, and may be nearly the same size as the noise in the measurements [47]. Thus, the noise in the measurement is one order of magnitude greater for TAPS than for SM-3. In practice, the attenuation difference for pendulums of even a few per cent can be a source of a false rotational signal, especially in a region where the rotational component is small compared to the translational one [48]. In order to overcome this problem, various techniques have been proposed for obtaining more reliable results for seismic rotation measurements using TAPS, unfortunately with limited effectiveness. These include two-channel signal equalisation using a special procedure before measurement [49], a filtering procedure in the FFT domain [46] or the time domain [47] and a recorded data spline function approximation [50]. Currently, eight TAPSs are used by the Institute of Geophysics PAS, Poland. Despite the registration of rotational events during earthquakes [17,42,51] and seismic activity connected with artificial detonation in mine regions [52,53,54], TAPS does not meet the requirements for seismological (S1–S4) and engineering (E1–E4) applications, as can be seen from the data presented in Table 1 [55].

### 3.2. Rotational Seismometers Using Pairs of Classical Geophones

**Figure 4:**
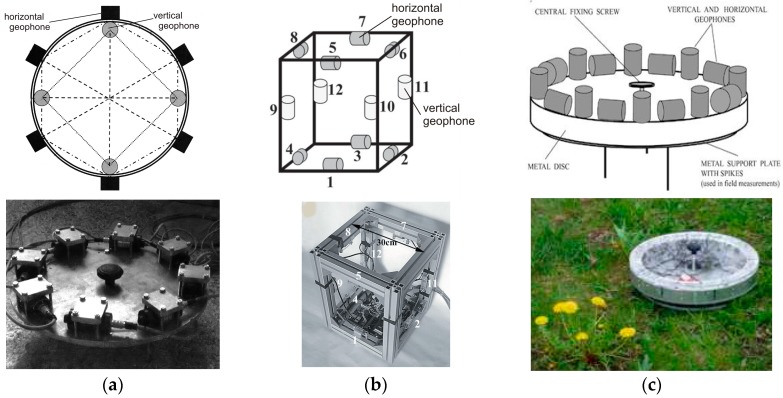
Schematic diagrams of the Rotaphones and general views: (**a**) schematic diagram of 3DOF prototype I [56] and view of prototype II [58]; (**b**) 6DOF prototype II [63]; (**c**) Rotaphone-D [61].

In 2009, Brokešová et al. constructed a practical example of an indirect rotational seismometer based on commercially available geophones, which they called the “Rotaphone” [56,57]. This is a set of geophones mounted on a rigid base either horizontally or vertically. The three-degree-of-freedom (3DOF) prototype consisted of 10 (four vertical and six horizontal) geophones (LF-24, Sensor Nederland B.V., Leiden, Netherlands) mounted around a metal disc of diameter 0.25 m and spaced regularly, as shown in Figure 4a [56,57]. A modified system contained only horizontal geophones, arranged in four diametrical pairs, to measure solely the vertical rotation rate *Ω_z_* (Equation (4)) and two horizontal components of the ground translational velocity [58].

The subsequent system, shown in Figure 4b, was named the six-degree-of-freedom (6DOF) system, and this contained 12 geophones mounted at the edges of a rigid tube with distance 0.3 m. This version had increased sensitivity due to its different configuration, low-noise geophones (SM-6, Sensor Nederland B.V.) and better analogue/digital (A/D) converters [59,60]. Finally the constructors of the 6DOF system returned to the initial scheme using mounted sensors around a disc, this time with 16 (eight horizontal and eight vertical) SM-6 geophones around disc with a separation of the paired geophones of 0.4 m, as shown in Figure 4c. This was named the Rotaphone-D [61]. The separating distances were chosen to correlate with a specific wavelength of interest. Due to the rigidity of the frames used, the components of rotation rate are calculated as [56]:

(4) $$\Omega_{x}=\frac{\partial v_{z}}{\partial y}=-\frac{\partial v_{y}}{\partial z},\;\Omega_{y}=\frac{\partial v_{x}}{\partial z}=-\frac{\partial v_{z}}{\partial x},\;\Omega_{z}=\frac{\partial v_{x}}{\partial y}=-\frac{\partial v_{y}}{\partial x}$$

where *x*, *y*, *z* are Cartesian coordinates, as shown in Figure 1, and *v*_i_ is a suitable time derivative of the displacement components measured by geophone.

Since both vertical and horizontal sensors are used, the rotation rate is determined by more than one geophone pair; this allows for a very precise in situ calibration of the geophones and improves the signal/noise ratio of both translation and rotation, so that the rotation rate can be calculated more accurately. A summary of data for the Rotaphone devices described above is presented in Table 1. These devices have been used to record many tens of seismic events both induced by natural sources (weak earthquakes with measured rotation in order of 10^−6^ rad/s) [57,58,62,63] and anthropogenic sources (blasts with measured rotation in order of 10^−3^ rad/s) [58,60,63]. Regarding requirements (S1–S4) and (E1–E4), the 6DOFs are close to fulfilling the requirements for seismological applications; however their frequency ranges are still too narrow, and they should be treated as short-period systems.

## 4. Rotational Seismometers for Direct Measurement of Rotation

In general, there are three different technologies for constructing this type of rotational seismometer: mechanical, electrochemical and optical.

### 4.1. Rotational Seismometer Using Mechanical Sensor Technology

**Figure 5:**
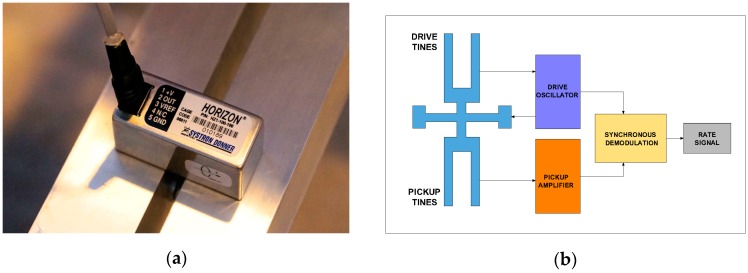
The Horizon™ MEMS angular rate sensor: (**a**) general view of the HZ1-100-100; (**b**) scheme of operation.

Mechanical systems are based on MEMS. Originally, the technique was developed for manufacturing integrated accelerometers for airborne applications. Since the suspended mass is very small in this case, the Q factor is required to be very high for the Brownian noise to be acceptably low [7]. Based on this idea, a company called Systron Donner Inertial (Concord, CA, USA) produced the Horizon™ [64], a compact, high reliability, solid-state angular rotation sensor (Figure 5a). The operation scheme of this device is shown in Figure 5b. The main elements are piezoelectric quartz tines, driven by an oscillator to vibrate at a precise amplitude, causing the tines to move forward and away from one another at a high frequency [65]. This vibration causes the drive fork to become sensitive to the rate of angular motion about an axis parallel to its tines. An applied rate of rotation causes a sine wave of torque to be produced in the tines of the sensor, resulting from the oscillating torque of the drive tines at this frequency. Electrical output signals are produced by the pickup amplifier as the pickup tines respond to the oscillating torque by moving in and out of plane.

These signals are amplified and converted into a DC signal proportional to the rate using a synchronous switch which responds only to the desired rate signals. The main parameters for the wide-range device, the HZ1-200-100, are summarised in Table 2. The manufacturer recommends using commercially available high-accuracy dynamic signal acquisition modules such as DT9837 (DataTranslation Inc., Marlboro, MA, USA), which combined with QuickDAQ software provides an easy way to store data and carry out analysis. From the data presented in Table 2, it can be seen that this can be used only as an additional device for laboratory investigation of engineering applications in rotational seismology, for example that in [66]. Its main advantages are its small size, power consumption and high clip level.

### 4.2. Rotational Seismometers Using Electrochemical Sensor Technology

**Figure 6:**
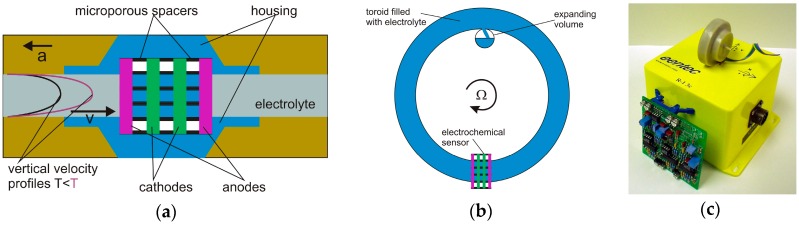
The electrochemical rotational seismometer: (**a**) schematic diagram of the MET transducer [40]; (**b**) a rotational sensor mechanical system design [71]; (**c**) the Eentec R-1 [71].

These electrochemical devices use a fluid as an inertial mass; the motion of the fluid is detected using multilayer platinum electrodes with a spacing of a few tenths of millimetre according to the scheme shown in Figure 6a [40]. The fluid is an ion-rich electrolyte and is free to move. At both ends of the channel, an elastic diaphragm allows for fluid motion. When a DC voltage is applied to the electrodes, it produces an ion concentration gradient between them. Due to the conductivity of the electrolyte, the bias voltage and its associated current produce an ion concentration gradient only between the electrodes. As the system is accelerated by a ground motion, the fluid flows relative to the electrodes and this yields a change in current, proportional to the fluid velocity and to the ion concentration; this is known as the molecular electronic transfer (MET) technique [70]. This transducer is essentially of the velocity type [7], in which the symmetric arrangement of the electrode pairs improves the transducer linearity, which may be further linearised and shaped by a feedback loop. This technique is especially appropriate for pure rotational seismometers, the operation of which is shown in Figure 6b [71]. The sensor has a toroidal channel filled with electrolyte. When the sensor rotates, the liquid is forced through the sensor MET placed across the channel, converting liquid motion into electrical output. The expansion volume is used to compensate for the expansion of the liquid due to temperature. Several models of such seismometers can be found on the company’s website (e.g., RSB-20 from PMD Scientific Inc., Weatogue, CT, USA (www.pmdsci.com), R-1 and R-2 from Eentec, Kirkwood, MO, USA (www.eentec.com), METR-01, METR-03, METR-11, METR-13 from R-sensors LLC, Moscow, Russia (www.r-sensors.ru), and the R-2 from AST LLC, Moscow, Russia (www.seismometers.ru)). Even though the manufacturers claim that the seismometers are rugged and are suitable for portable field use because they have no springs, hinges or moving mechanical parts (except the fluid), there is limited information about field testing of these devices [39,40,72,73]. For this reason, Table 2 presents the main parameters for devices manufactured by Eentec R-1 [68] and the prototype R-2, probably developed in cooperation with the small Russian company AST [69].

As can be seen, the Eentec rotational seismometers have parameters which almost match those to expected for seismological and engineering applications. However, fulfilling the S4 and E4 requirements is still problematic. A sequence of tests carried out between 2006 and 2010 showed reasonable results for higher frequencies. Testing for linear and cross-axis sensitivity for R-1 showed that its linear sensitivity of 6 × 10^−5^ rad/s/(m/s^2^) and 2% cross-axis sensitivity are conservative at the maximum value [72] and were twice as high as expected [73]. The same doubt remains about the quality of the calibration, especially in the lower (<1 Hz) frequency range [39], since the frequency response does not have a flat shape, and at frequencies above 1 Hz the dynamic range is 80 dB instead of the claimed value of above 110 dB [72]. For this reason, it has been suggested that better resolution of one order of magnitude for the recording of weak earthquakes is required [40]. Finally, deviations from the nominal value of 27% and 18% in the scale factor values for R-1 and R-2 in a temperature range of 20 °C to 50 °C have been measured [40], giving rise to the suggestion that the liquid-based technology requires further improvement for reliable field measurements.

Despite the above reservations, installed R-1 rotational seismometers have for instance recorded several hundred local earthquakes and two explosions in Taiwan [74]. The largest peak rotational rate recorded at the HGSD station (up to early 2008) was from an earthquake with magnitude 5.1 at 13:40 UTC 23 July 2007. The peak rotational rate was of 0.63 × 10^−3^ rad/s for the vertical component with a dominant frequency band of about 2.5–5.5 Hz.

### 4.3. Rotational Seismometers Using Optical Sensor Technology

**Figure 7:**
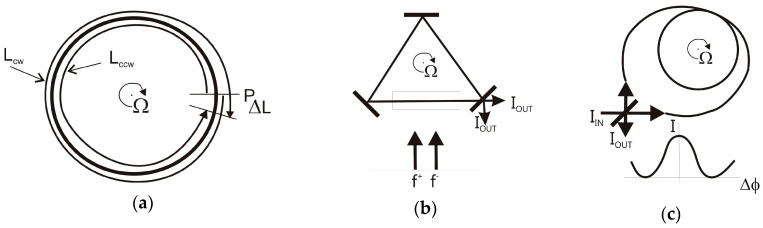
The Sagnac effect in a circular ring interferometer rotating with respect to an inertial frame of reference: (**a**) interferometric systems for its detection; (**b**) active method in the ring-laser approach; (**c**) passive method in a fibre-optic interferometer approach. Notation: L_cw_, L_ccw_—distances in clockwise and counterclockwise directions; I_IN_, I_UOT_—intensities of input and output beams respectively [77].

The optical rotational seismometer uses an optical gyro configuration, which operates based on the Sagnac effect (more precisely, the von Laue-Sagnac effect) [75]. This effect can be observed in any loop interferometer, as shown in Figure 7a, since the optical path length difference *∆L* experienced by light propagating in opposite directions along the interferometer which is rotating with rate **Ω** is [76]:

(5) $$\Delta L=\frac{4A}{c_{0}}\Omega ,$$

where **A** is the vector of the geometrical area enclosed by the wave path, *c*_0_ is the velocity of light in a vacuum and **Ω** is the rotation vector. It can be seen that the Sagnac effect depends on the scalar product of two vectors (**A**, **Ω**), showing that the system detects only rotational components with an axis perpendicular to the geometrical area enclosed by the wave path; this axis can be positioned freely according to this area [76]. In general, the distance *∆L* generated by the Sagnac effect is extremely small; for instance, the Earth’s rate of rotation (0.26 rad/h) gives a magnitude of *∆L* equal to 9.7 × 10^−15^ m for an area of 10^−2^ m^2^. Hence, ring laser and fibre-optic type systems (Figure 7b,c) are technical implementations of the loop interferometer for appropriate detection of distances of this magnitude or lower.

**Figure 8:**
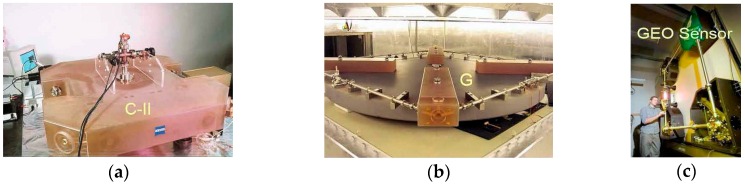
The ring laser rotational seismometer [84]: (**a**) C-II, horizontally installed; (**b**) G, horizontally installed; (**c**) GEO, vertically installed.

The ring-laser set-up for the measurement of *∆L* is the loop interferometer, which includes an optical amplifier within the resonator [78]. This type of amplifier enables laser oscillation at f^q^ along the (q = +) and (q = −) directions within the resonator (lower part of Figure 7b). In the presence of rotation **Ω**, the frequency difference *∆f* is given by:

(6) $$\Delta f=f^{+}-f^{-}=\frac{4A}{\lambda P}(n,\Omega ),$$

where *λ* is the optical wavelength of the laser oscillator, **n** is the normal vector to the laser beam plane and *P* is the perimeter enclosed by the beam path. The ring-laser approach using a He-Ne amplifier [79] was the first successful ring-laser gyroscope (RLG) and is now being used in a number of civilian and military navigation systems. The implementation of this type of system for seismological research has been proposed in various systems including the C-II [80] and GEO ring-lasers [81] in Christchurch, New Zealand, and the G-ring laser in Wettzell, Germany [82] (Figure 8). These have two major advantages for applications in seismic studies compared to the other seismometers discussed above, since they measure absolute rotation with respect to the local universe, and they do not depend on accelerated masses. In particular, this last property ensures an extremely wide dynamic range of operation, from a few 10^−6^ Hz for geophysical signals up to more than 10 Hz, as obtained from regional earthquakes [83]. Since the G-ring laser is at present the system with the best signal-to-noise performance, its parameters are included in Table 3 for comparison with other optical rotational seismometers.

Since ring-laser rotational seismometers are optimised for the detection of very weak rotational signals at extremely low frequencies, these are stationary devices mounted in stabilised underground environmental conditions such as temperature, pressure and low vibration conditions. For the above reasons, they generally fulfil only the S1, E1 and S4 requirements. However, for the last two decades they have been sufficient for practical use in the detection of rotation events in both strong and weak earthquakes [83,85,86,87].

The other device, and probably the most promising, is an optical rotational seismometer based on the fibre-optic gyroscope (FOG) [94]; its basic configuration is schematically illustrated in Figure 7c. For a fibre of length *L* wound in a coil of diameter *D*, a phase shift is produced between counter-propagating light of magnitude *∆φ*, given by [95]:

(7) $$\Delta \varphi =\frac{2\pi LD}{\lambda_{0}c_{0}}\Omega ,$$

where Ω is the rotation component along the axis perpendicular to the fibre-optic loop, and *λ*_0_ is the wavelength of the light in a vacuum. In other words, the sensitivity of the Sagnac interferometer in this approach is enhanced not only by increasing the diameter of the physical sensor loop but also by increasing the total length of the used fibre.

**Figure 9:**
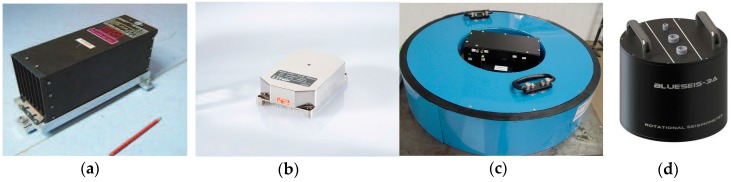
Rotational seismometers based on FOG: (**a**) LCG-demonstrator [40]; (**b**) μFORS-1 [89]; (**c**) AFORS-1; (**d**) BlueSies-3A [93].

An approach using a classical FOG [48,96] was the first successful application of this type of system for seismological research. The next generation of these systems was the FORS-II, installed in the Ojcow Observatory, Poland [97] for the investigation of rotational events, which had a resolution of 4.3 × 10^−8^ rad/s @ 1 Hz for an optimised sensor loop radius and optical fibre length. Limited information can be found in the literature on other applications of the commercial FOG as a rotational seismometer. Bernauer et al. [40] described a laboratory investigation of the temperature stability of the LCG-Demonstrator based on LCR-100 AHRS (Northrop Grumman LITEF GmbH, Freiburg im Breisgau, Germany), shown in Figure 9a, with parameters presented in Table 3. Within a temperature range of between 20 °C and 50 °C, these authors observed no scale factor error, whereas the Allan deviation of the seismometer indicated an amplitude-modulated white noise in periods from 0.1 to 500 s. A power consumption of 25 W and the rather low sensitivity of the LCG-Demonstrator restrict this device mainly to rotational engineering applications in the authors’ opinion. Similar conclusions can be drawn from a laboratory investigation and a field test of the μFORS-1 device at a wind generator [88] (Figure 9b with parameters in Table 3). The main reason for this limited application is probably related to the fact that commercial FOGs have integrated electronics which are optimised to measure angle changes but not rotational rates. In order to avoid this problem, new systems with special electronics have been proposed. The first is our autonomous fibre-optic rotational seismograph (AFORS-1), which is characterised in Table 3. This device, shown in Figure 9c, has been used continuously in the Książ Observatory, Poland since 21 July 2010. It records seismic events which are stored on the spot together with data from two sets of TAPS for comparison of their recordings [90,91,98,99] as well as sending this to a FORS-Telemetric Server via GPS (see http://fors.m2s.pl with login and password: AFORSbook). The main advantage of the AFORS-1 [99] is the possibility of using a full system remote control via the internet. However its main disadvantages are a frequency band which is too low, and a maximum detectable rate of a few mrad/s which limits parameters for AFORS application in the seismological area of interest. In view of this, the next rotational seismometer, known as the fibre-optic system for rotational events and phenomena monitoring (FOSREM) (the laboratory investigation of which is summarised in Section 5), has been proposed [100] as a device for seismological and engineering applications.

The iXBlue (Cedex, France) was presented at a meeting in the first half of 2016 [92,93]. The prototype includes a broadband and high-grade three-component fibre-optic rotational seismometer BlueSeis-3A; its parameters are listed in Table 3 and a general view is shown in Figure 9d. An analysis of the parameters claimed for this device shows that it is a rotational seismometer for low self-noise and broadband measurement. The manufacturer has announced extensive laboratory testing for later this year, and this device may be available by next year.

## 5. FOSREM as a System for Seismological as Well as Engineering Applications

Experience connected with the use of AFORS and the requirements for engineering applications provided the basis for the realisation of a new device, FOSREM [100], as a rotational seismometer for seismological and engineering applications that fulfils all the requirements described in Section 2.

### 5.1. Construction, Operation and Main Parameters of FOSREM

**Figure 10:**
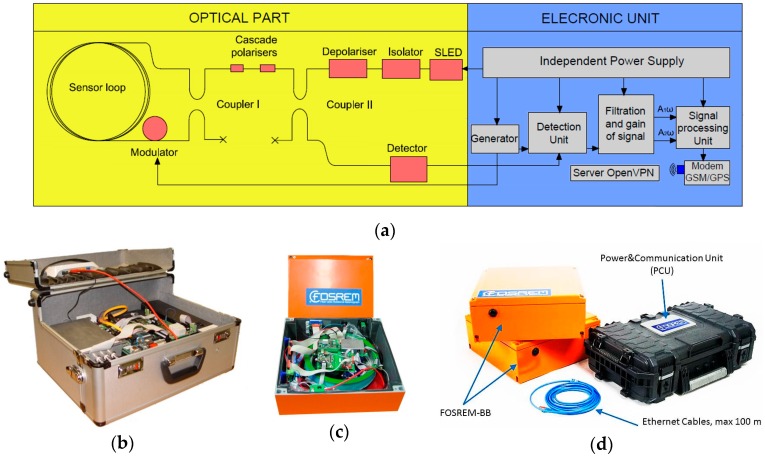
FOSREM: (**a**) general scheme of the system; (**b**) view of FOSREM-SS; (**c**) view of FOSREM-BB; (**d**) multi-sensor synchronous measuring system based on FOSREM-BB.

Figure 10 shows FOSREM, which was previously described in detail [101]. This device consists of two parts: optical and electronic. The optical part is constructed using a so-called minimum configuration of FOG [94], which can detect an extremely low rotation rate by protection of the Sagnac effect as a unique non-reciprocal effect in the system. This is obtained using a common input-output way (two couplers) with the selection of a single-mode operation (a kind of optical fibre used) and the same polarisation (cascade polarisers) for two interacting beams. Since the sensor loop contains a 5.000 m standard single-mode fibre (SMF-28) wound on a 0.215 m diameter spool we used an additional depolariser for system operation on depolarised light in the sensor loop. The total losses in the optical part in the range of 16 dB for the 10 mW light source (SLED) reaches theoretical sensitivity 2 × 10^−8^ rad/s/Hz^1/2^. The electronic unit calculates and records rotational data through the use of open-loop synchronous detection in a digital form using a 32-bit DSP. This involves specific electronic solutions using signal processing to directly determine the component of rotation according to a previously developed approach [102] using the following formula [101]:

(8) $$\Omega =S_{o}\cdot \arctan[S_{e}\cdot u(t)]=S_{o}\cdot \arctan[S_{e}\cdot \left(A_{1\omega }/A_{2\omega }\right)]$$

where *S_e_*, *S_o_* are the electronic and optical constants of the system, and *A*_1ω_ and *A*_2ω_ are the first and second amplitudes of the harmonic output signal [*u*(t)].

It should be emphasised that the applied measurement method provides a completely different technical approach than that used in FOG. In FOG, the parameter of interest is the angle direction obtained by applying a suitable integration procedure to the rotation rate recorded using the Sagnac effect. If a commercial FOG system is used for the construction of a rotational seismometer, the rotation rate is a suitable time derivative of the output signal, which introduces additional errors. The FOSREM electronic unit protects of the rotation rate detection derived in Equation (7), and is thus free from such errors. Moreover, FOSREM uses a specific averaging method for initial data recording, for the elimination of drift phenomena [97,102]. Data storage and system control are realised over the internet using a connection between FOSREM and GSM/GPS or Ethernet. Experimental research was carried out using two systems, as shown in Figure 10b,c, in which the FOSREM-BB with an improved analogue detection unit is designed to work as a multi-sensor synchronous measuring system including three FOSREM-BB systems and a Power and Communication Unit (PCU) (see Figure 10d). The connection provides data transmission and power supply over a single standard STP cable within a distance of 100 m. The four multi-sensor systems can operate in a single network, transferring data to a central cloud-based system via the internet.

The FOSREM enables measurement of only the rotational component, over a wide range of signal amplitudes (from 2.06 × 10^−8^ rad/s/Hz^1/2^ to a few rad/s) as well as in a wide frequency band from DC to 328.12/*n* Hz (*n* = 1, …, 128). Dimensions are 470 mm × 360 mm × 230 mm for FOSREM-SS and 360 mm × 360 mm × 160 mm for FOSREM-BB; the weight is below 10 kg, and power supply is 230VAC + 14.4V/20Ah Li-On battery (12 h for the operational system) for FOSREM-SS and PoE 48V from PCU for FOSREM-BB. These aspects, combined with the remote control of the electronic module possible via the internet [93] mean that the FOSREMs are portable and autonomous devices. Beside these differences in weight, size and power management, the two FOSREMs have additional differences in maximum rotation rate measurement; this was optimised for FOSREM-BB according to its patent application [102].

The accuracy of both systems has been evaluated based on the measurement of the defined constant angular velocity of the Earth in Warsaw, Poland (Ω_E_ = 4.45 × 10^−5^ rad/s for φ = 52°20′). The obtained accuracies are in the range 3 × 10^−8^ rad/s to 1.6 × 10^−6^ for the abovementioned frequency bandpass [101]. Moreover, FOSREMs are stable during cooling and heating processes within a temperature range of 0 °C to 50 °C with temperature sensitivity of the scale factor <0.03%/°C [101].

### 5.2. Recording Strong Rotational Motion with a New Set-Up Using Earthquake Simulations

**Figure 11:**
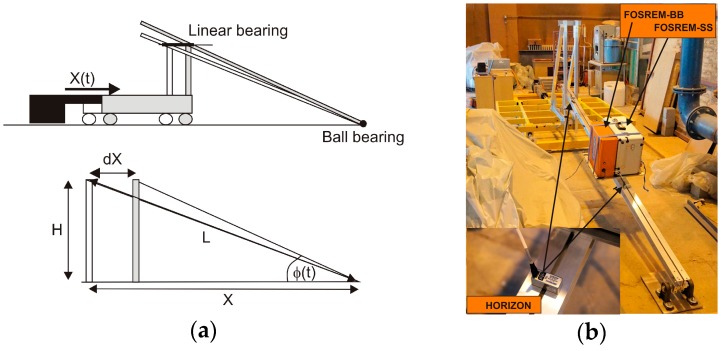
Modified shaking table: (**a**) scheme (top image) and trigonometric dependence for Equation (9) (bottom image: H = 0.5 m, L = 3.7 m); (**b**) general view of shaking table with mounted FOSREMs and HZ1-100-100.

A typical shaking table (2.0 m × 2.0 m platform with a maximum load of 1000 kg, moving back and forth in the horizontal plane X(t) with power supplied by a dynamic actuator) was used in the experiments carried out at the Gdansk University of Technology, Poland. This table allows the generation of horizontal shaking which is a realistic simulation of that occurring during earthquakes. For testing of FOSREM, the table was modified using an additional part, as shown in Figure 11a. This configuration allows for the movement of the beam in the vertical plane during the horizontal movement of the table platform, which introduces a change in the angle φ(t) between the ground and the beam supported by the ground. The platform of the seismic table was controlled by introducing a normalised record of accelerations. Using the Simpson method, the velocity of the table *v*(t) from the installed accelerometer 333B52 (PCB Piezoelectronics Inc., Depew, NY, USA) was calculated. These data were then used in order to determine the introduced rotation Ω for the sensors installed on the moving beam using the following relation (notation as for Figure 11a):

(9) $$\Omega ={\frac{d\phi \left(t\right)}{\mathrm{dt}}}_{|\phi \left(t\right)={\mathrm{ctan}}^{-1}\left(\frac{X-dX}{H}\right)}=\frac{1}{1+{\left(\frac{X-dX}{H}\right)}^{2}}{\frac{dX}{dt}}_{|dX\ll X}=\frac{H}{\left[H^{2}+{\left(\frac{X}{H}\right)}^{2}\right]H}v\left(t\right)=\frac{H}{L^{2}}v\left(t\right)=0.0365v\left(t\right)\left[\frac{rad}{s}\right]$$

**Figure 12:**
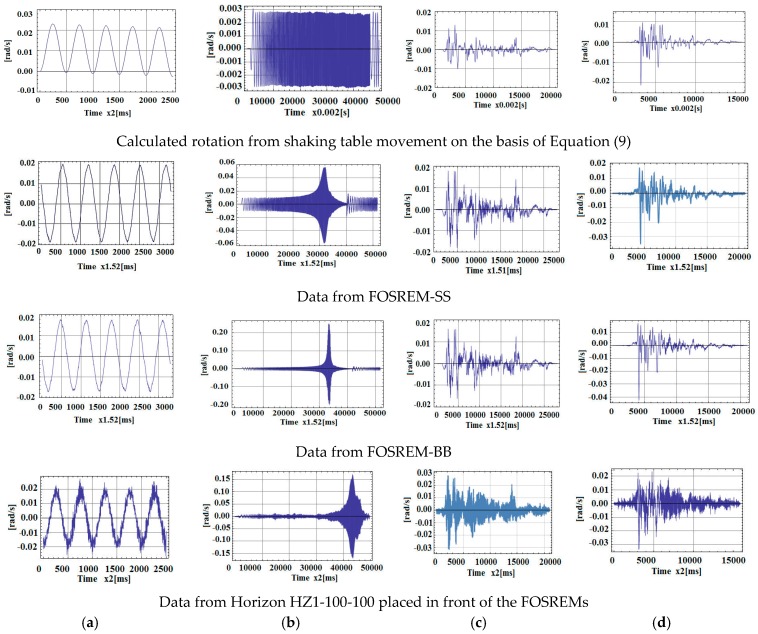
Data obtained by various devices for (**a**) sine excitation; (**b**) sweep sine from 0.25 Hz to 10 Hz; (**c**) El Centro earthquake; (**d**) Loma Prieta earthquake.

In order to carry out the test, based on this digitised data, the two FOSREMs were mounted on the beam together with two rotational sensors of type Horizon HZ1-100-100 (parameters as listed in Table 2 for HZ1-200-100, without the clip level limited to 1.74 rad/s and a scale factor deviation of twice as large [65]) as shown in Figure 11b. The test used a sinusoidal excitation (Figure 12a), a sweep sine excitation in the frequency band 0.25 to 10 Hz (Figure 12b), and simulation of the real seismic earthquakes registered in California on 18 May 1940 in El Centro (Figure 12c) and on 17 October 1989 at Loma Prieta (Figure 12d). The first line in Figure 12 presents the rotation rate forced by a shaking table evaluated on the basis of the installed accelerometer; the following three lines in Figure 12 represent the data for FOSREM-SS, FOSREM-BB and Horizon, respectively.

**Figure 13:**
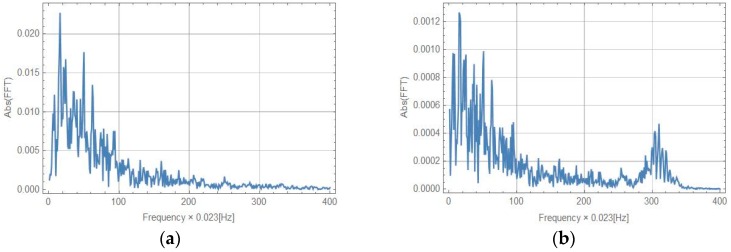

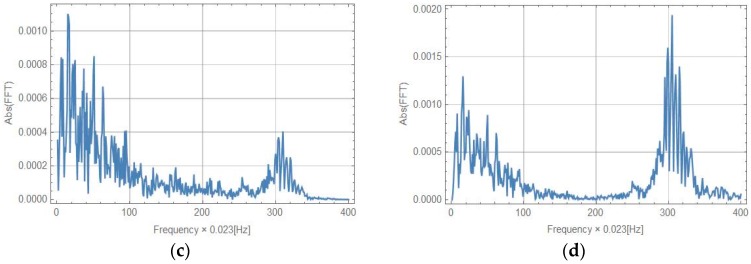
Spectrum characteristics for sweep sine excitation: (**a**) rotation from accelerometer PCB 333B52; (**b**) FOSREM-SS; (**c**) FOSREM-BB; (**d**) HZ1-100-100.

The results indicate a good correlation between the real rotation used for the driven table (line 1 in Figure 12) and recorded by FOSREM-SS and FOSREM-BB. Both FOSREMs showed similar recorded signals. The presented data also clearly show that the commercially available single-axis rotational sensor HORIZON has lower sensitivity than the FOSREMs, which is reflected in the more illegible trace of the recorded signal, especially for lower values of signal amplitude. In addition, the location of this sensor close to the linear bearing influenced an observed additional noise connected with the operation of the bearing. Moreover, for a sweep sine excitation, the nonlinear characteristics of the rotation rate induced in the set-up were observed. This is connected with the resonance characteristics of the beam with the mounted seismometers, with a natural frequency of about 7.15 Hz as indicated in the spectrum characteristics presented in Figure 13. The advantage of this phenomenon is the possibility of reaching high values of the maximum rotation rate detected by FOSREM-BB in the range of 0.25 rad/s. From the data presented for FOSREM-SS in Figure 12 for sweep sine excitation, it can be seen that this device achieves results of above 0.06 rad/s only, which correspond with the abovementioned incorrect operation of analogue signal processing unit in FOSREM-SS. The other device, FOSREM-BB, is free from this disadvantage.

It can be concluded that the parameters presented for FOSREM-BB meet all the requirements for a rotational motion sensor for both seismological and engineering applications; thus, this sensor is the most promising for rotational seismology applications.

## 6. Conclusions

The main objective of this paper was to review the existing solutions in terms of rotational seismometers from a practical point of view. This approach assumes unique requirements for rotational motion sensors for seismological and engineering applications. However, there is no explicit consensus on these requirements, and the formulation of these on the basis of the literature as put forward in Section 2 of this work is, to the authors’ knowledge, the first time this has been carried out.

In this paper, three tables are presented which give the main parameters of the existing devices. These should be treated as initial points for future discussion, since common parameters for all the types of rotational seismometers examined here do not yet exist. This is likely to present a serious difficulty in the future development of these devices. We consider here the need for a new approach to the definition of parameters as well as a standard methodology for their investigation, since rotational seismometers have a completely different principle of operation from classic seismometers. The review presented here clearly shows that devices which use mass inertia as their main physical principle of operation are likely to be limited in their future utility due to their limited sensitivity, dynamic range and operational band, and this is mainly related to their nonlinear frequency characteristics.

Arising from the above fundamental constraint, are devices which use light for their operation. The von Laue-Sagnac effect is currently a useful basis as a physical principle for construction of the rotational seismometer, as one can see from the parameters presented by existing devices. The main advantage of this type of sensor is its complete insensitivity to linear motion and its direct measurement of rotational speed.

The development of the optical gyroscope nearly half a century ago offers an excellent technological and technical solution for the construction of an optical rotational seismometer. Despite the incredible sensitivity of ring-laser rotational seismometers, their dimensions, power consumption, and environment instability mean that such devices are best suited for stationary research into fundamental geophysical phenomena.

The review presented here shows that fibre-optic rotational seismometers are the most attractive option, since their parameters can meet all the requirements of the various areas of interest within rotational seismology. Unfortunately, as can be observed from their limited applications, the direct application of the commercially available FOGs does not fulfil these requirements, since FOGs are optimised for monitoring angle changes rather than rotation rate. In view of this, new types of devices are required, and BlueSeis-3A and FOSREM are the first of these. FOSREM, presented in this paper, fulfils all the technical requirements for rotational motion detection, in both seismological observatories and in engineering constructions. It guarantees a wide range of the detected signal amplitude up to 10 rad/s, as well as a wide range of frequencies, from DC up to 328.12 Hz. Experimental investigation indicates that FOSREM has an accuracy in the range 3 × 10^−8^ to 1.6 × 10^−6^ rad/s in the abovementioned frequency bandpass, and in practice detects rotation with an amplitude of 0.25 rad/s. It is a remotely controlled sensor which is portable and works autonomously. Additionally, the use of cloud system by FOSREM allows the integration of dozen of sensors in a worldwide network, each transferring data to the central cloud-based system. The data can be viewed and analysed from anywhere in the world via the internet. The authors believe that the further application of FOSREM in the investigation of rotational seismology effects will contribute to the provision of interesting and useful data.

## Figures and Tables

**Table 1:** Overview of rotational seismometers using indirect measurement (only the most important parameters are listed).

Parameter	Unit	TAPS [55]	Rotaphone		
			3DOF [62]	6DOF [62]	D [61]
Frequency range	Hz	7 × 10^−1^–50 ^(1),(2)^	1–100 ^(2)^	2–60 ^(2)^	2–80 ^(2)^
Sampling frequency	Hz	100	250	250	250
Sensitivity ^(3)^	rad/s	1 × 10^−7^	1.67 ×10^−8^	2.16 × 10^−9^	3.77 × 10^−9^
Maximum rate	rad/s	1 × 10^−1^	1 × 10^−2^	2.87 × 10^−1^	3.17 × 10^−2^
Dynamic range	dB	120	100	120	120
Paired sensor spacing	m	0.28	0.30	0.30	0.40
Operating temperature	°C	−10–45	−20–40	−20–40	−40–100 ^(4)^
Weight	kg	15	4.5	9.5	15.3
Dimensions [L × W × H]	mm	450 × 180 × 350	250 ^(5)^ × 10	350 × 350 × 430	445 ^(5)^ × 112
Sensors: [pcs × type]		2 × SM-3	8 × LF-24	12 × SM-6	16 × SM-6
Natural frequency	Hz	4.5	1	4.5	4.5
A/D converter:	type	Sigma-Delta	2 × AD16021	4 × Tedia	1 × EE & S
dynamic	Bit	26	21	28	24
range	V	±10	±5	±2.5	±1 or ±2.5
GPS receiver and antenna		Stationary system	Garmin GPS 18 (mobile)		
Software:	type	Own	Own	Own	Own
output format		miniSEED	RotaCal	RotaCal	RotaCal

^(1)^ Modified according to recorder MK-6 by IG PAS; ^(2)^ The instrument generally operates in a high-frequency range (above the natural frequency of the sensors used); ^(3)^ Understood as an expression for the smallest signal that can be resolved ([7], p. 79); ^(4)^ Data for geophone SM-6; ^(5)^ Disc diameter.

**Table 2:** Overview of rotational seismometers using direct measurement (only the most important parameters are listed).

Parameter	Unit	HZ1-200-100 [67]	R-1 [68]	R-2 [69]
Axial		uniaxial	triaxial	triaxial
Sensitivity ^(1)^	rad/s/√Hz	4.4 × 10^−4^	1.2 × 10^−7^	0.6 × 10^−7^
Clip level ^(2)^	rad/s	3.49	0.10	0.40
Dynamic range	dB	78	110	117
Frequency band	Hz	>60	0.05–20	0.03–50
optional extended		n/a	0.03–50	0.01–100
Scale factor ^(3)^	V/rad/s	0.57(±2%)	50	50
optional		n/a	2 × 10^2^	5–2 × 10^2^
Operating temperature	°C	−40 to +71	−15 to +55 (extended −45 to +55)	
Output signal	V	+0.5 to +4.5	±5, ±2.5	±20 differential
Calibration (S.F. deviation from 20/22 °C)	%/°C	<0.08	<0.03	Internal calibration electronics
Shock survival	g	200	200	200
Power supply	VDC	8–12	9–14	9–18
Supply current	mA	<20	20	30
Power consumption	W	0.24	0.28	0.54
Weight	kg	<0.06	1.0	1.5
Dimensions [L × W × H]	mm	58.3 × 25.3 × 25.3	120 × 120 × 90	120 × 120 × 100
NEMA rating		4	4	Waterproof (submersible)
Software	type	Own	Own	Own

^(1)^ For unambiguous comparison with data in Table 1, this is output noise for SNR = 1, also defined as resolution @ 1 Hz in [rad/s]; ^(2)^ Identical to the maximum rate in Table 1; ^(3)^ Understood as the gain of the instrument ([7], p. 79).

**Table 3:** Overview of optical rotational seismometers with RLG and FOG configurations (only the most important parameters have been listed).

Parameter	Unit	G-Ring [85]	μFORS-1 [88,89]	LCG ^(1)^ [40]	AFORS-1 [90,91]	BlueSeis-3A [92,93]
Axial		uniaxial	uniaxial	triaxial	uniaxial	triaxial
Sensitivity ^(2)^	rad/s/√Hz	9 × 10^−11^	3 × 10^−5^	6.3 × 10^−7^	4 × 10^−9^	2 × 10^−8^
Maximum Rate	rad/s	1	17.5	No data	6.4 × 10^−3^	0.1
Dyn. Range	dB	280	115	No data	124	135
Freq. Band	Hz	0.003–10	No data	DC–100	0.83–106.15	DC–100
S. F. Error ^(3)^	%/°C	Not observed	≤0.05(1σ)	Not observed	No data	<0.01
Oper. Temp.	°C	Constant	−40 to 77	No data	−10 to 50	−10 to 50
Calibration		Needs	No data	Not needed	Remote	Not needed
Shock Survival	g	No data	250	10	No data	No data
Power Supply	VDC	high	±5, 3.3	24	12	24
Power Cons.	W	high	2.5	25	<24	<20
Weight	kg	No data	0.137	2.7	18	20
Dimensions [L × W × H]	mm	Area equal to 16 m^2^	22 × 73 × 58	278 × 102 × 128	700 diameter × 160	300 × 300 × 280
Ingress Protection		none	hermetically sealed		none	IP66
Sampling rate	Hz	4	5 to 1000	200	212	up to 200
Output format		No data	TIL/CMOS	miniSEED	miniSEED	miniSEED
Software	type	No data	No data	UDP Ethernet protocol	Web-based interface for configuration	

^(1)^ LCG-Demonstrator based on the LCR-1000 gyrocompass AHRS; ^(2)^ For unambiguous comparison with data in Table 1, this is output noise for SNR = 1 defined also as resolution @ 1 Hz in [rad/s]; ^(3)^ Defined also as the temperature sensitivity of scale factor.
